# A systematic review on tracheostomy decannulation: a proposal of a quantitative semiquantitative clinical score

**DOI:** 10.1186/1471-2466-14-201

**Published:** 2014-12-15

**Authors:** Pierachille Santus, Andrea Gramegna, Dejan Radovanovic, Rita Raccanelli, Vincenzo Valenti, Dimitri Rabbiosi, Michele Vitacca, Stefano Nava

**Affiliations:** Department of Life Science, Università degli Studi di Milano. Pulmonary Rehabilitation Unit, Fondazione Salvatore Maugeri, Istituto Scientifico di Milano-IRCCS, Via Camaldoli, 64-20138 Milan, Italy; Department of Pathophysiology and Transplantation, Università degli Studi di Milano, IRCCS Fondazione Cà Granda Ospedale Maggiore Policlinico, Via F. Sforza, 33-20122 Milan, Italy; Department of Biomedical Sciences for Health, Università degli Studi di Milano. Respiratory Unit, Policlinico San Donato IRCCS, Piazza E. Malan, 1-20097 San Donato Milanese, Italy; Unit of Maxillo-Facial Surgery. Ospedale San Paolo, Milano, Università degli Studi di Milano, Milano, Via A. di Rudinì, 8-20141 Milan, Italy; Pulmonary Rehabilitation Unit, Fondazione Salvatore Maugeri, Via Mazzini, 129- 26088 Lumezzane, Brescia, Italy; Alma Mater University Department of Clinical, Integrated and Experimental Medicine (DIMAS), Respiratory and Critical Care Unit, S. Orsola-Malpighi Hospital, Via Albertoni, 10-40138, Bologna, Italy; Dipartimento di Scienze della Salute, Università degli Studi di Milano. Pulmonary Rehabilitation Unit, Fondazione Salvatore Maugeri, Scientific Institute of Milan-IRCCS, Via Camaldoli, 64-20138, Milan, Italy

**Keywords:** Tracheostomy, Decannulation, Predictive score, Clinical score, Removal tube

## Abstract

**Background:**

Tracheostomy is one of the most common surgical procedures performed in critical care patient management; more specifically, ventilation through tracheal cannula allows removal of the endotracheal tube (ETT). Available literature about tracheostomy care and decannulation is mainly represented by expert opinions and no certain knowledge arises from it.

**Methods:**

In lack of statistical requirements, a systematic and critical review of literature regarding tracheostomy tube removal was performed in order to assess predictor factors of successful decannulation and to propose a predictive score. We combined 3 terms and a literature search has been performed using the Cochrane Central Register of Controlled Trials (CENTRAL); MEDLINE via Ovid SP; EMBASE via Ovid SP; EBSCO. Abstracts were independently reviewed: for those studies fitting the inclusion criteria on the basis of the title and abstract, full-text was achieved. We included studies published from January 1, 1995 until March 31, 2014; any sort of review and expert opinion has been excluded by our survey. English language restriction was applied. Ten studies have been considered eligible for inclusion in the review and were analysed further.

**Results:**

Cough effectiveness and ability to tolerate tracheostomy tube capping are the most considered parameters in clinical practice; other parameters are taken into different consideration by many authors in order to proceed to decannulation. Among them, we distinguished between objective quantitative parameters and semi-quantitative parameters more dependent from clinician’s opinion. We then built a score (the Quantitative semi Quantitative score: QsQ score) based on selected parameters coming from literature.

**Conclusions:**

On our knowledge, this review provides the first proposal of decannulation score system based on current literature that is hypothetical and requires to be validated in daily practice. The key point of our proposal is to give a higher value to the objective parameters coming from literature compared to less quantifiable clinical ones.

## Background

Tracheostomy is one of the most frequent procedures applicated in intensive care unit (ICU) patients: about 10% of patients requiring more than 3 days of mechanical ventilation are expected to undergo tracheostomy [1]. Furthermore, in the last years the development of less invasive surgical techniques allowed tracheostomy to be safely performed at patient’s bedside and at present this techniques are growing more and more because of the increased number of patients requiring difficult or prolonged weaning from endotracheal tube (ETT), due to aging and severe comorbidities [2]. Moreover, possibility of tracheotomy for prevention and better clinical management of ventilator-associated pneumonia was recently evaluated [3].

As a result, the correct management of tracheostomized patients and defined procedures for decannulation grew into a more important clinical issue.

### Physiological modifications after tracheostomy

In the following chapter we will discuss the main physiological implications for breathing after tracheostomy. In scientific literature, authors usually compare tracheostomy to endotracheal intubation. In our purpose, we intended to compare changes during tracheostomy to spontaneous breathing and the most relevant topics are:

*Airflow resistance* - Resistance of anatomically normal airways is firstly conditioned by upper airways (up to 80% during nose breathing and 50% during mouth breathing). Therefore, tracheostomy should virtually reduce flow resistance bypassing the respiratory upper airways. Actually, data from scientific literature state the contrary, as the presence of tracheotomy tube, during spontaneous breathing, reduces airway radius leading to increased flow resistance and, of course, increased work of breathing (WOB) [4, 5].

This is according to Poiseuille equation, as the resistance to gas flow through a tube varies inversely with the internal diameter of the tube (in particular, to the 4th power of the radius of the tube when flow is laminar).

Secondly, secretions (stimulated even more by the presence of the tube) still play an important role: adhering to the inner lumen of the tube, they can lead to significant luminal narrowing, according to Wilson et al. [6], 15% of tubes are shrunk three sizes; other experiences from literature ultimately come to the same conclusions [7, 8].

It has been supposed that the inner diameter of a smaller tracheostomy tube may induce increased flow resistance and WOB, if compared to spontaneous breathing. The first data from in vivo studies appeared recently by Valentini et al. trying to define the effect of smaller tracheostomy tube sizes on diaphragm effort. They recorded diaphragm pressure- time product per min (PTPdi/min), tension-time index of the diaphragm (TTdi), and the ratio of respiratory rate to tidal volume (f/VT) for 2 different tracheotomy tube, with an inner diameters 8 mm and 6.5 mm. The use of a smaller diameter resulted in an increase of diaphragmatic effort, decrease of VT and an increase of intrinsic PEEP. In conclusion, the authors assessed that, especially in hard to wean tracheostomized patients, the use of a tracheostomy tube of small size can lead to alterations of some weanibility parameters, otherwise normal with greater size tubes [9].

In addition, a single physiologic study by Criner et al. reported that airways resistance and work of breathing resulted higher with the tube in place, during spontaneous breathing, when compared to breathing after decannulation [10].

*Humidification and heating* - Normally upper airways play a fundamental function of heating and humidification of inspired air. In tracheostomized patients, the air bypasses the nasopharyngeal cavity and enters directly the tracheobronchial tree; a system of artificial humidification is needed. In the absence of adequate humidification, the epithelium of the trachea is involved in a gradual inflammatory process resulting in squamous metaplasia and, consequentially , impairment of ciliary function and increased risk of respiratory infection [11].

### Physiological modifications after decannulation

While scientific literature has better investigated tracheotomy effects on respiratory physiology, data about respiratory mechanisms after decannulation are almost totally lacking. Chadda et al. studied respiratory parameters in nine neuromuscular patients after that the upper airways were confirmed to be free of obstructions by fiberoptic bronchoscopy. In this experience, decannulation resulted in an increase of the tidal volume and carbon dioxide partial pressure due to an increase of the dead space and work of breathing [12].

However, the argument is still controversial. In a study of few years ago, Dellweg et al. did not find a significant difference in WOB in favour of tracheostomy when compared to mouth breathing. Decannulation increases or decreases airways resistance depending from case to case and, in particular, from the morphology of the upper airways [13].

To our knowledge, there are only few papers on physiological modifications after decannulation; experts have differing opinion on this topic and so further research and trials are desirable.

### Weaning from tracheostomy – problem generation

Place a tracheostomy enables discharging the patient from ICU to a rehabilitation unit [14]. However, despite decannulation is not risk-free, there is evidence of benefits for tracheostomy tube removal. The tracheostomy tube may cause inflammation and stenosis or excessive cough and may impair swallowing by preventing the physiological trachea’s elevation against the epiglottis in order to prevent aspiration of food or secretions [15].

Furthermore, upper airways are excluded from breathing. At last, in most cases tracheostomized patients are unable to speak; aphonia worsens patient’s quality of life and slows down the recovery process, often leading to anxiety and depression.

A number of clinically important early and late complications have been evaluated, including granulation tissue, tracheal stenosis, tracheomalacia, tracheo-esophageal fistula, ventilator-associated pneumonia and aspiration. According to literature, chronic tracheostomy in severe Chronic Obstructive Pulmonary Disease (COPD) patients is associated with a higher frequency of exacerbations requiring antibiotic treatment [16]. The clinical relevance of these complications may lead to death and most of patients weaned from mechanical ventilation through tracheostomy should undergo early decannulation.

As it is hard to schematize the approach for decannulation, we decided to perform the following revision of literature, with the aim of supporting or retracting current statements. Which are then current issues in managing tracheostomy? How does evidence-based medicine handle the process of weaning from tracheostomy tube? Nowadays clinical habit derives from physiopathological knowledge, from personal experience and from professional practice: there is limited evidence in literature regarding specifically decannulation processes. Little is known about how clinicians decide to decannulate patients. Which are the criteria for choice?

## Methods

### Criteria for considering studies for this systematic review

**Figure 1:**
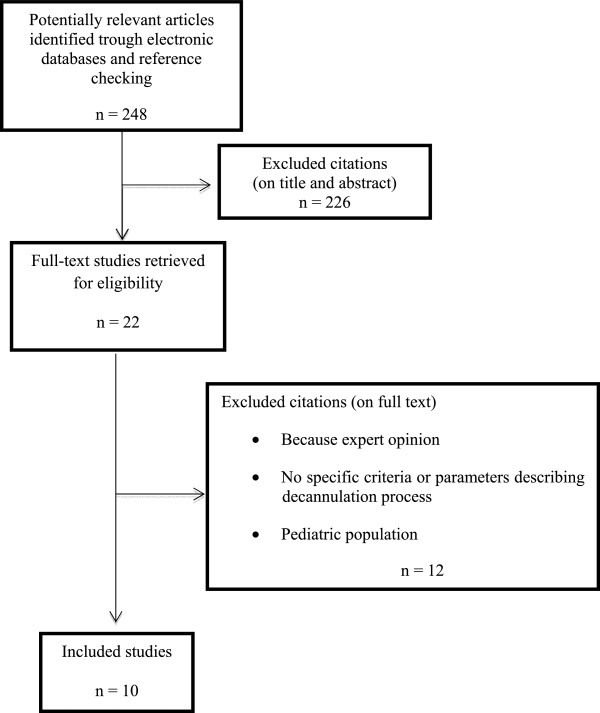
**Flow diagram of the search process.** The number of references initially identified through each database was 248. References were usually excluded for more than one reason by a two consecutive steps.

A literature search has been performed using the Cochrane Central Register of Controlled Trials (CENTRAL); MEDLINE via Ovid SP; EMBASE via Ovid SP; EBSCO. Research included, but was not limited to, 3 keywords (tracheostomy, decannulation, weaning). Abstracts were independently reviewed: for those studies fitting the inclusion criteria on the basis of the title and abstract, full-text was achieved. Reference lists were also examined for any additional relevant studies not identified through the former search. We included studies published from January 1, 1995 until March 31, 2014. Such studies examined a population more than 18 years old, who received tracheostomy for any clinical reason, excluded neuromuscular diseases, and hospitalized in any ward (ICU, rehabilitation wards, etc.); any sort of review and expert opinion has been excluded by our survey. Including English language restriction was applied. A flow chart diagram of the search strategy and study selection is provided in Figure 1.

## Results

The database search yielded 248 citations published between January 1995 and September 2012 (with duplicates removed). 226 articles were excluded on the title and abstract. The full-text of potentially relevant articles was achieved for further evaluation. The final number of papers taken into account is 10. Figure 1 shows the flow-chart explaining how many citations were excluded from the analysis and for what reasons. After analyzing the selected literature, we decided for a systematic and critical revision of literature on decannulation, due to lack of statistical requirements for a meta-analysis. Specifically, in Table 1, primary and – when present – secondary outcomes proposed by each author have been evaluated for each study.

**Table 1:** **Primary and secondary outcomes evaluated for each study**

Authors	Primary outcomes	Secondary outcomes
Bach et al. 1994 [19].	• PCF ≥ 160 L/min	• VC
		• Age
Ceriana et al. 2003 [20].	• Clinical stability (no active infection and hemodynamic stability)	
	• Absence of psychiatric disorders	
	• Effective cough (MEP ≥ 40 cmH2O)	
	• PaCO2 < 60mmHg	
	• Adequate swallowing (evaluated by gag or blue dye test)	
	• Absence of tracheal stenosis (evaluated by endoscopy)	
Stelfox et al. 2008 [26].	• Ability to tolerate tube capping (24h vs. 72h)	• Oxygenation (SaO2 95% with FiO2 0,3 vs. 0,5)
	• Cough effectiveness (strong vs. weak)	• RR (18 bpm vs. 28 bpm)
	• Secretions (scan thin vs. moderate thick)	• Swallowing (enteral nutrition via gastric tube and nothing p.o. vs. enteral nutrition via gastric tube and jelly and pudding)
	• Level of consciousness (alert vs. drowsy but arousable)	
		• Indication for tracheostomy (pneumonia vs. COPD)
		• Difficulty of intubation (easy vs. difficult)
		• Comorbidities (no significant comorbidities vs. end-stage renal disease)
		• Age (45 yo vs. 75 yo)
Stelfox et al. 2009 [27].	• Ability to tolerate tube capping (24h vs. 72h)	• Oxygenation (SaO2 95% with FiO2 0,3 vs. 0,5)
	• Cough effectiveness (strong vs. weak)	• RR (18 bpm vs. 28 bpm)
	• Secretions (scan thin vs. moderate thick)	• Swallowing (enteral nutrition via gastric tube and nothing p.o. vs. enteral nutrition via gastric tube and jelly and pudding)
	• Level of consciousness (alert vs. drowsy but arousable)	
		• Indication for tracheostomy (pneumonia vs. COPD)
		• Difficulty of intubation (easy vs. difficult)
		• Comorbidities (no significant comorbidities vs. end-stage renal disease)
		• Age (45 yo vs. 74 yo)
Budweiser et al. 2011 [23].	• Ability to tolerate tube capping > 24h/48h	• Serum creatinine
		• Duration of former intubation and tracheostomy
	• Oxygenation	
	• Age	
O’Connor et al. 2009 [24].	• Shorter permanence at acute facility	• Ability to tolerate tube capping
		• Cough effectiveness
Marchese et al. 2010 [28].	• Stability or respiratory conditions (dyspnea, RR, SaO2, PaO2, PaCO2, pH)	
	• Effective cough	
	• Indication for tracheostomy (underlying disease)	
	• Effective swallowing	
	• No or mild hypercapnia (PaCO2 level in stable state)	
Choate et al. 2008 [21].	• Cough effectiveness	
Leung et al. 2003 [22].	• Indication for tracheostomy (unstable or obstructed airways vs. others)	
Tobin et al. 2008 [25].	• Ability to tolerate tube capping > 24h	
	• Cough effectiveness (no need of suctioning)	
	• Setting of cure (intensivist-led tracheostomy team vs. others)	

PCF = Peack Cough Flow; VC = Vital Capacity; MEP = Maximal Expiratory Pressure; PaCO2 = Partial pressure of carbon dioxide in the blood; RR = Respiratory Rate; SaO2 = ratio of oxyhaemoglobin to the total concentration of haemoglobin present in the blood; FiO2 = fraction of inspired oxygen concentration.

Studies targeting different outcomes from the ones predictors in decannulation were excluded, although they considered tracheostomy tube removal of enrolled patients as a step. Surveys were also included. Contextually, papers exploring new and/or experimental techniques for the assessment of risk in trachestomy tube removal were excluded [17, 18]. Such exclusion was operated because the proposed approaches - such as oscillometry impedance measurement or upper airway resistance measurement – are difficult to apply in every setting and are not easily reproducible in different contexts. Nevertheless in this context innovative techniques, such those before reported, could probably be useful to improve decannulation procedure in future.

First of all, it must be noted that literature is mainly made of expert opinions and international surveys; the few reported clinical trials are mainly descriptive (retrospective and prospective); because of technical and ethical problems, Randomized Clinical Trials (RCTs) are totally lacking.

It can be noticed from the data that the population taken into consideration is heterogeneous for age, comorbidities and causes of tracheostomy; it must also be emphasized that studies take in consideration both acute/reversible pathologies and chronic conditions at different levels of severity (for instance, Budweiser et al. [2] considers prolonged weaning patients with persistent respiratory failure). Secondly, recommendations differ between clinicians who work in acute facilities and those who work at chronic wards and between respiratory therapists and physicians.

In our analysis cough effectiveness and ability to tolerate tracheostomy tube capping are the most frequent criteria used by clinicians in order to predict successful decannulation. In others studies a different importance is also given to parameters such as oxygenation and capnia, level of consciousness and neurological state, age, swallowing, quantity and quality of secretions, duration of mechanical ventilation, stability of haematic gases (PaO2 and PaCO2), aetiology of respiratory failure and comorbidities.

Bach et al. experienced in a population of patients with respiratory failure due to different aetiologies some parameters that can predict the success of the tracheostomy decannulation. The most important predictive factor is PCF (Peak Cough Flow), in particular at least 160 L/min; other important factors are also VC (Vital Capacity) and age [19]. Ceriana e al. proposed a prospective study to decide whether to remove tracheotomy in long-term mechanically ventilated patients with respiratory failure from different causes. By the use of a decisional flowchart based on clinical and physiological parameters, they were able to remove tracheotomy cannula in almost 80% of patients with spontaneous breathing without major clinical complications. Principal parameters considered were patient's ability to remove secretions, swallowing function, absence of psychiatric diseases, possibility of reaching spontaneous breathing, and amount of respiratory space [20].

Choate K and Barbetti J conducted a prospective descriptive study of consecutive patients who received a tracheostomy in ICU. Of the 823 decisions for decannulation, there were 40 episodes of failed decannulation, representing a failure rate of 4.8%. The main reason for decannulation failure was sputum retention and ineffective cough [21].

Lastly, in a retrospective study on patients requiring tracheostomy in ICU, Leung and his team identified risk factors that would indicate a low likelihood of early decannulation. The indications for tracheostomy were prolonged mechanical ventilation, tracheobronchial toilet or risk of aspiration and unstable or obstructed airways. They concluded that the only indicator for early decannulation is tracheostomy insertion and other patient related variables are not significant. [22].

The usefulness of a tracheostomy retainer (TR) and the predictors of successful decannulation were also evaluated by Budweiser and his team. In percutaneously tracheostomized patients with prolonged weaning, the use of a TR seems to facilitate the weaning process. Furthermore, also the duration of spontaneous breathing prior to decannulation, age and oxygenation predict the risk of recannulation [23].

O’Connor et al. retrospectively examined the process of decannulation following tracheostomy in patients transferred to a long-term care hospital for weaning from prolonged mechanical ventilation. Decannulation was successful in 35% of patients and main factors taken in account were ability to tolerate tube capping and cough effectiveness. Patients who failed decannulation had an earlier placed tracheostomy tube and had also a shorter permanence at the acute facility compared with patients who were decannulated [24].

Similar results were described by Tobin and his team; the authors also demonstrated that an intensivist-led tracheostomy team is associated with quicker decannulation time and a shorter hospitalization [25].

Stelfox et al. performed a cross-sectional survey on 200 physicians and respiratory therapists with expertise in the management of tracheostomized patients to characterize state-of-art about tracheostomy decannulation practice and to define their opinions about factors influencing these practices. Clinicians rated patient level of consciousness, ability to tolerate tracheostomy tube capping, cough effectiveness, and secretions as the most important factors in the decision to decannulate a patient. Decannulation failure was defined as the need to reinsert an artificial airway within 48 hours (45% of respondents) to 96 hours (20% of respondents) of tracheostomy removal. In clinical scenarios, clinicians who worked in chronic care facilities (30%) were less likely to recommend decannulation than clinicians who worked in rehabilitation (53%) or acute care (55%) facilities (p = 0.015).

In a similar North American survey by same authors, ability to tolerate capping, secretions, cough effectiveness, and level of consciousness as the most important factors in the decannulation decision [26, 27]. Another national one-year survey evaluating clinical criteria and systems for performing decannulation was conducted by Marchese et al. in this population main clinical criteria chosen for decannulation are: stability of respiratory conditions before and after closure of tracheostomy tube, effective cough, underlying diseases and ability to swallow. Moreover, laryngo-tracheoscopy has been considered in order to exclude contraindications to decannulation [28].

## Discussion

### A new proposal

The parameters considered in literature and also reported in Table 1 are worth of deeper analysis.

First of all, we identified two different types of parameters: Quantitative parameters: objective, described by means of numerical values and provided with cut-off (such as, ability to tolerate tube capping > 24h); Semi-quantitative parameters: objective, but however not be easily described by mean of a numerical value (as, for instance, swallowing function). Our purpose is to suggest a clinical score including all parameters – both quantitative and semiquantitative –considered by available scientific literature in order to evaluate the feasibility of tracheostomy tube removal.

In our idea, objective quantitative parameters shall be taken into greater account in decisional process. Other parameters should instead be evaluated, when it is possible, according to a binary system (e.g. dysphagia yes/no).

Our intent is to give a high score (e.g. 20 pts) to patients fitting quantitative objective parameters. When such requirements are missing, the score of each single parameter will be 0 pts. This choice is to underline the fundamental importance we assign to those parameters, being the most frequent measurable ones taken in consideration by current scientific literature; in lack of those parameters, scientific evidence seems to predict a negative outcome for decannulation.

In the second place, our clinical score must comprehend as well semi-quantitative objective clinical parameters (e.g. dysphagia and secretions) and subjective parameters (e.g. clinician experience). In our systematic review the utility of bronchoscopy before and during decannulation appears as an important tool that should be considered in clinical practice.

Most of these parameters respond to the need for a straightforward and binary evaluation (yes/no = 5pts/0pts); a smaller numerical value has been assigned to them in order to lower their weight on the overall score versus objective parameters.

With reference to the two different types of parameters taken into account, we suggest to name the proposed score “QsQ score”, that is “Quantitative semi Quantitative score”. In Table 2 such approach is summarized.

**Table 2:** **QsQ score: Quantitative and semiquantitative parameters**

Parameter	Cut-off	Missing	Fitting
	***Objective quantitative parameters – Main criteria***		
Cough	MEP ≥ 40 cmH2O	0	20
	PCF > 160 L/min		
Tube capping	≥24 h	0	20
	***Semi-quantitative parameters – Minor criteria***		
Level of counsciousness	Drowsy/Alert	0	5
Secretion	(thick vs. thin)	0	5
Swallowing	Impaired/Normal	0	5
Capnia	paCO2 < 60 mmHg	0	5
Patent airway	Tracheal stenosis < 50% seen by bronchoscopy	0	5
Age	<70	0	5
Indication for tracheostomy	Others/Pneumonia or airway obstruction	0	5
Comorbidities	Present (≥1) or None	0	5

This hypothetical score have the objective quantitative parameters, named ‘major criteria’, and semi-quantitative or subjective parameters, named ‘minor criteria’. For the proposed interpretation and clinical application see the text in Discussion section.

MEP = Maximal Expiratory Pressure; PaCO2 = partial pressure of carbon dioxide in the blood; RR = Respiratory Rate; SaO2 = ratio of oxyhemoglobin to the total concentration of hemoglobin present in the blood; FiO2 = fraction of inspired oxygen concentration.

We underline that the twenty point and five points threshold has been chosen *a priori*, without previously performing an experimental validation; our aim is to highlight the role of objective parameters taken in consideration by most of the studies.

### Score: hypotheses and interpretations

We suggest an hypothetical score, that requires discussion and a prospective validation study. For a practical use we will name objective quantitative parameters ‘major criteria’, and semi-quantitative or subjective parameters ‘minor criteria’. If all main criteria are satisfied, regardless of minor criteria, decannulation with high probability of positive outcome can be assumed.

If only one of the two major criteria is satisfied, a careful evaluation of minor criteria should be required, assuming a good probability of positive outcome when the majority of minor criteria is satisfied. The same probability category reported above could be applied if, in lack of major criteria, all of minor criteria are satisfied. Finally, if none of the major criteria and less than three minor criteria are satisfied, a low probability of positive outcome can be assumed.

## Conclusions

Tracheostomy decannulation represents one of the most important problems in the clinical and home care management of patients which undergo tracheostomy. No validated and specific pathway is followed when performing a decannulation and this process is left to the clinical expertise. Considering this, we hypothesized a clinical score, named QsQ, to help clinicians in choosing decannulation timing. We underline that this score has never been validated in clinical real life and therefore we suggest evaluating QsQ in further clinical trials to validate it.

## Abbreviations

PCF: Peak Cough Flow
VC: Vital Capacity
MEP: Maximal Expiratory Pressure
PaCO2: partial pressure of carbon dioxide in the blood
RR: Respiratory Rate
SaO2: Ratio of oxyhemoglobin to the total concentration of hemoglobin in blood
FiO2: Fraction of inspired oxygen concentration
QsQ: Quantitative and semiquantitative score.

## References

[1] Durbin CG (2010). Tracheostomy: why, when, and how?. Respir Care.

[2] MacIntyre NR, Epstein SK, Carson S, Scheinhorn D, Christopher K, Muldoon S (2005). Management of patients requiring prolonged mechanical ventilation: report of a NAMDRC consensus conference. Chest.

[3] Terragni PP, Antonelli M, Fumagalli R (2010). Early vs late tracheotomy for prevention of pneumonia in mechanically ventilated adult ICU patients: a randomized controlled trial. JAMA.

[4] Cavo J, Ogura JH, Sessions DG, Nelson JR (1973). Flow resistance in tracheotomy tubes. Ann Otol Rhinol Laryngol.

[5] Heffner JE (2003). Tracheotomy application and timing. Clin Chest Med.

[6] Wilson AM, Gray DM, Thomas JG (2009). Increases in endotracheal tube resistance are unpredictable relative to duration of intubation. Chest.

[7] Epstein SK, Ciubotaru RL (1996). Influence of gender and endotracheal tube size on preextubation breathing pattern. Am J Respir Crit Care Med.

[8] Mehta S, Heffer MJ, Maham N, Nelson DL, Klinger JR, Levy MM (2010). Impact of endotracheal tube size on preextubation respiratory variables. J Crit Care.

[9] Valentini I, Tonveronachi E, Gregoretti C, Mega C, Fasano L, Pisani L, Nava S (2012). Different tracheotomy tube diameters influence diaphragmatic effort and indices of weanability in difficult to wean patients. Respir Care.

[10] Criner G, Make B, Celli B (1987). Respiratory muscle dysfunction secondary to chronic tracheostomy tube placement. Chest.

[11] Epstein SK (2005). Anatomy and physiology of tracheostomy. Respir Care.

[12] Chadda K, Louis B, Benaissa L (2002). Physiological effects of decannulation in tracheostomized patients. Intensive Care Med.

[13] Dellweg D, Barchfeld T, Haidl P, Appelhans P, Kohler D (2007). Tracheostomy decannulation: implication on respiratory mechanics. Head Neck.

[14] Scheinhorn DJ, Chao DC, Hassenpflug MS, Gracey DR (2001). Post-ICU weaning from mechanical ventilation: the role of long-term facilities. Chest.

[15] Christopher KL (2005). Tracheostomy decannulation. Respir Care.

[16] Clini E, Vitacca M, Bianchi L, Porta R, Ambrosino N (1999). Long term tracheostomy in severe COPD patients weaned from mechanical ventilation. Respir Care.

[17] Franke KJ, Nilius G, Morgenstern S (2011). Removal of the tracheal tube after prolonged mechanical ventilation: assessment of risk by oscillatory impedance. Respiration.

[18] Gao C, Zhou L, Wei C, Hoffman MR, Li C, Jiang JJ (2008). The evaluation of physiologic decannulation readiness according to upper airway resistance measurement. Otolaryngol Head Neck Surg.

[19] Bach JR, Saporito LR (1994). Indications and criteria for decannulation and transition from invasive to noninvasive long-term ventilatory support. Respir Care.

[20] Ceriana P, Carlucci A, Navalesi P, Rampulla C, Delmastro M, Piaggi G, De Mattia E, Nava S (2003). Weaning from tracheotomy in long-term mechanically ventilated patients: feasibility of a decisional flowchart and clinical outcome. Intensive Care Med.

[21] Choate K, Barbetti J, Currey J (2009). Tracheostomy decannulation failure rate following critical illness: a prospective descriptive study. Aust Crit Care.

[22] Leung R, MacGregor L, Campbell D, Berkowitz RG (2003). Decannulation and survival following tracheostomy in an intensive care unit. Ann Otol Rhinol Laryngol.

[23] Budweiser S, Baur T, Jorres RA, Kollert F, Pfeifer M, Heinemann F (2012). Predictors of successful decannulation using tracheostomy retainer in patients with prolonged weaning and persisting respiratory failure. Respiration.

[24] O'Connor HH, Kirby KJ, Terrin N, Hill NS, White AC (2009). Decannulation following tracheostomy for prolonged mechanical ventilation. J Intensive Care Med.

[25] Tobin AE, Santamaria JD (2008). An intensivist-led tracheostomy review team is associated with shorter decannulation time and lenght of stay: a prospective cohort study. Crit Care.

[26] Stelfox HT, Crimi C, Berra L, Noto A, Schmidt U, Bigatello LM, Hess D (2008). Determinants of tracheostomy decannulation: an international survey. Crit Care.

[27] Stelfox HT, Hess DR, Schmidt UH (2009). A North American survey of respiratory therapist and physician tracheostomy decannulation practices. Respir Care.

[28] Marchese S, Corrado A, Scala R, Corrao S, Ambrosino N (2010). Tracheostomy in patients with long-term mechanical ventilation: a survey. Respir Med.

[CR29] The pre-publication history for this paper can be accessed here:http://www.biomedcentral.com/1471-2466/14/201/prepub

